# Synthetic cannabinoids use in a sample of opioid-use disorder patients

**DOI:** 10.3389/fpsyt.2022.956120

**Published:** 2022-08-03

**Authors:** María Alías-Ferri, Manuela Pellegrini, Emilia Marchei, Roberta Pacifici, Maria Concetta Rotolo, Simona Pichini, Clara Pérez-Mañá, Esther Papaseit, Robert Muga, Francina Fonseca, Magi Farré, Marta Torrens

**Affiliations:** ^1^Addiction Research Group, IMIM-Hospital del Mar Medical Research Institute, Barcelona, Spain; ^2^Department of Psychiatry and Legal Medicine, Universitat Autònoma de Barcelona, Cerdanyola del Vallés, Spain; ^3^National Centre on Addiction and Doping, Istituto Superiore di Sanità, Rome, Italy; ^4^Department of Clinical Pharmacology, Hospital Universitari Trias i Pujol and Institut de Recerca Germans Trias i Pujol (HUGTiP-IGTP), Badalona, Spain; ^5^Department of Pharmacology, Therapeutics and Toxicology, Universitat Autònoma de Barcelona, Cerdanyola del Vallès, Spain; ^6^Department of Internal Medicine, Hospital Universitari Germans Trias i Pujol and Institut de Recerca Germans Trias i Pujol (HUGTiP-IGTP), Badalona, Spain; ^7^Department of Medicine, Universitat Autònoma de Barcelona (UAB), Cerdanyola del Vallès, Spain; ^8^Addiction Program, Institute of Neuropsychiatry and Addictions, Hospital del Mar, Barcelona, Spain; ^9^Department of Medicine and Life Sciences (MELIS), Universitat Pompeu Fabra, Barcelona, Spain

**Keywords:** cannabis, synthetic cannabinoid, opioid use disorder, new psychoactive substances, urine sample analysis

## Abstract

Cannabis is the most widely consumed illegal drug in the world and synthetic cannabinoids are increasingly gaining popularity and replacing traditional cannabis. These substances are a type of new psychoactive substance that mimics the cannabis effects but often are more severe. Since, people with opioids use disorder use widely cannabis, they are a population vulnerable to use synthetic cannabinoids. In addition, these substances are not detected by the standard test used in the clinical practice and drug-checking is more common in recreational settings. A cross-sectional study with samples of 301 opioid use disorder individuals was carried out at the addiction care services from Barcelona and Badalona. Urinalysis was performed by high-sensitivity gas chromatography-mass spectrometry (GC-MS) and ultra-high-performance liquid chromatography-high –resolution mass spectrometry (UHPLC-HRMS). Any synthetic cannabinoid was detected in 4.3% of the individuals and in 23% of these samples two or more synthetic cannabinoids were detected. Among the 8 different synthetic cannabinoids detected, most common were JWH-032 and JWH-122. Natural cannabis was detected in the 18.6% of the samples and only in the 0.7% of them THC was identified. Several different synthetic cannabinoids were detected and a non-negligible percentage of natural cannabis was detected among our sample. Our results suggest that the use of synthetic cannabinoids may be related to the avoidance of detection. In the absence of methods for the detection of these substances in clinical practice, there are insufficient data and knowledge making difficult to understand about this phenomenon among opioid use disorder population.

## Introduction

Cannabis is the most used illicit drug worldwide, with an estimated 200 million users (approximately 4% of the world's population) between the ages of 15 and 64 in 2019 (1). In Europe, it is also the most widely used drug, with a prevalence of daily use of 1.8% in the general population and 10.3% in individuals between 15 and 24 years of age, respectively (2).

In recent years, synthetic cannabinoids (SCs) have emerged as substitutes for natural cannabis as they are cannabinoid types 1 and 2 receptor agonists similar to tetrahydrocannabinol (THC), the main psychoactive component of cannabis, which is a partial agonist (3). The most common products containing SCs are smoking mixtures, e-liquids, and infused paper (4). Some effects of SCs intoxication are loss of consciousness, respiratory depression, and behavioral alterations such as aggression or self-injury (5). Over the years, different generations of SCs have appeared, each time showing a higher potency than THC, making them attractive to some consumers (3, 6). Recently, a fourth generation of SCs has been described, which could cause serious damage to health based on its pharmacological and toxicological activity (7). The effects experienced may differ between SC users, as in the case of cannabis use, including feelings of euphoria, relaxation, or even paranoia (5); however, they are usually more intense than those experienced with natural cannabis (8).

SCs were first synthesized in 1970 in an attempt to find new analgesics for pain treatment, but it was not until the 2000s that they appeared in the market (9). The popularity of these compounds was boosted in 2004 by the emergence of a new product called “Spice” (4). A few years later, in 2008, the first cases of poisoning related to SCs use were reported (4), some of which resulted in fatal overdoses (10). Since 2008 to date, the European Union Early Warning System controls 209 different SCs; thus, SCs are the largest group of new psychoactive substances (NPS) monitored (2, 4).

Cannabis is commonly used by patients with opioid use disorder (OUD) (11), which points to this population as a potential consumer of SCs. Other SC users include regular cannabis users, people who experiment with new drugs (e-psychonauts), increasingly vulnerable groups, such as high-risk users (4, 12), and men aged between 13 and 59 with a history of polydrug use who consider SCs a good alternative to cannabis (13). Since SCs are not detected in the standard toxicological urine tests used in clinical practice, they can be easily used by individuals for pleasure and enjoyment (13).

Notably, the prevalence of SC use is <1% in the European general population, but this is higher if we focus on subpopulations such as young adults or psychiatric patients, especially those with psychosis (4). The national survey among the Spanish general population shows a prevalence of SC use of the 0.6% and increases to 1.2% in the group aged between 25 and 34 years (14).

OUD individuals have not been the focus population for studying these substances, which are more likely to be present in recreational settings, such as music festivals or raves, or among e-psychonauts. In the present study, we investigated the use of natural and synthetic cannabis in an OUD population from Barcelona through urinalysis.

## Materials and methods

### Study design and participants

A secondary analysis from a cross-sectional study of 301 OUD individuals was conducted. The samples belong to two collections: one from identified patients from whom sociodemographic and clinical data were collected, and the other from anonymous patients with no data collected. Patients were recruited at addiction care facilities at Hospital del Mar (Barcelona) and Hospital Universitari Germans Trias i Pujol (Badalona) in Spain, from February 2019 to March 2020 and from July to October 2020, respectively. Due to the impact and changes in the functioning of addiction care services during the COVID-19 pandemic, no samples were collected from March 13^th^ until July 6^th^, 2020.

Participation in the study was voluntary, and urine samples were collected from each participant. All participants, as an inclusion criterion, have been diagnosed with OUD by a psychiatrist/psychologist according to the fifth edition of the Diagnostic and Statistical Manual of Mental Disorders (15), participation in an opioid agonist treatment program, and >18 years of age. None of the eligible participants were excluded from the study.

The study was approved by the local Ethical Committee of Clinical Research of the Parc de Salut MAR (CEIC-PSMAR number: 2018/8138/I) and the Hospital Universitari Germans Trias i Pujol (CEIC-HUGTiP number: PI-18-126).

Other details of the participants and methods can be found in previous publications that did not focus on SCs (16, 17).

###  Urine analysis

Urine samples (9 ml) from recruited individuals were collected and stored at −20 °C (in Nunc CryoTubesTM) until analysis. Sample preparation involved a liquid-liquid extraction and urinalysis was performed using two different validated methodologies. Ultra-high-performance liquid chromatography high-resolution mass spectrometry (the full scan MS and fragmentation data-dependent MS/MS) was processed by Thermo Scientific TraceFinder™ software. The built-in database, mass spectral library of over 1,000 compounds of which more than fifty SCs (naphthoylindoles, phenylacetylindoles, indazole carboxamides, tetramethylcyclopropylindoles), retention times, isotope pattern matching, elemental composition determinations are used to identify and confirm drugs and metabolites in the analyzed samples. The matching threshold to establish LOIs (limit of identification) was set at 80%. Moreover, mzCloud Mass Spectral Library was also used as mass spectra international library for peak identification (Advanced Mass Spectral Database; www.mzcloud.org). In gas chromatography, the full scan data files were processed by an AgilentWorkstation (Agilent Technologies). The mass spectra international library (NIST, National Institute of Standards and Technology research library) was used for peaks identification (18).

### Data analysis

Frequency-based descriptive analysis was carried out using SPSS (version 22.0; SPSS Inc., Chicago, IL, USA).

## Results

A total of 301 urine samples were collected and analyzed. Although more than 50% of the samples were collected anonymously, subjects were part of an opioid agonist treatment program, the sociodemographic characteristics of patients in this program indicate that 68% of the patients were men and the mean age was 52 years (range: 22–77 years old).

The SCs and cannabis derivatives detected in these samples are shown in Table 1.

**Table 1:** Cannabis-related substances and metabolites detected among opioid use disorder urine samples (*N* = 301).

**Substances and metabolites detected**	**N (%)**
Samples positive to SCs^*^	**13 (4.3)**
JWH-018	1 (0.3)
JWH-032	4 (1.3)
JWH-122 or JWH-122 N-4-hydroxypentyl / JWH-122 N-5- hydroxypentyl	4 (1.3)
JWH-200	2 (0.7)
JWH-209	2 (0.7)
JWH-210 or JWH-210 N-4-hydroxypentyl / JWH-210 N-5- hydroxypentyl	2 (0.7)
RCS-8	1 (0.3)
UR-144 or UR-144 N-5-hydroxypentyl	1 (0.3)
Samples positive to natural cannabinoids^*^	**56 (18.6)**
Cannabidiol	-
Cannabinol	-
THC	2 (0.7)
11-Nor-9-carboxy-THC (THC-COOH), cannabidiol, or cannabinol	56 (18.6)

* Samples can contain more than one SCs o natural cannabinoid.

Some SCs were detected in 13 (4.3%) urine samples, and 8 different substances of this type were identified. Among these, two or more SCs were found in three (23.07%) samples. The most detected NPS cannabinoid types were JWH-032 and JWH-122 in four (1.3%) cases each. JWH-018, RCS-8, and UR-144 were less common, present in only one (0.3) case each.

Natural cannabis was detected in 56 (18.6%) samples. In all cases, carboxy-THC, cannabidiol, or cannabinol were identified. THC was identified in only two (0.7%) of the samples.

## Discussion

We detected the use of SCs in individuals with OUD who were attending addiction care facilities in Barcelona and Badalona. Notably, cannabis use was widespread among the study participants with a Contrary to our results, previous studies in Finland and Germany did not find the presence of SCs in a similar population (19, 20). Furthermore, previous studies have investigated the prevalence of natural cannabis use in OUD populations. While some show a similar, although somewhat lower prevalence of 15% in positive urine samples (21), other studies show a higher prevalence of e.g., 58% (20) and 63% (22). Differences in the prevalence of cannabis use in this population could be explained by characteristics of the sample such as the country where the studies were conducted.

This study highlights the importance of investigating the consumption of SCs in this population, as there are limited studies on this topic. Along with this lack of knowledge, we found that polydrug use in the OUD population is widespread and often includes cannabis and cannabinoids among other substances (17, 23). Polydrug use is associated with an increased risk of relapse, fatal overdose, and suicidal ideation and attempts (23). Of note, SCs are one of the groups of NPS with the highest number of reported intoxications (24).

Human studies for the investigation of the clinical aspects of SCs are currently limited and usually focused on cases of intoxication or fatalities (25). Although, these are some reports of observational studies focused on pharmacological effects and fewer on the detection of these substances in addicted populations, such as OUD individuals, as is the case of the present study (26–28).

We hypothesized that there are several factors that could explain the increased use of cannabis and SCs, in recent years. First, the legalization of this substance in several countries has contributed to a lower perception of the risk of consumption in the population than the risk perception pre-legalization (1, 29). Another reason is the increase in the consumption of cannabinoids for therapeutic or medical purposes, which can lead to misuse and even abuse of these substances (5). Finally, the view that SCs are safer than other drugs and are a good alternative to natural cannabis indicates that these substances are potentially abused (30).

## Conclusion

We detected several types of SCs in patients with OUD in Barcelona and Badalona. Additionally, a non-negligible percentage of cannabis use was detected in our sample. These findings suggest that cannabis use is prevalent among patients with OUD and may be substituted by cannabinoid-like NPS to avoid detection in clinical tests. Since we do not have the instruments and protocols for NPS detection in clinical practice, knowledge about this phenomenon is very limited in this population. It would be interesting to continue this line of research to have more updated knowledge about the use of SCs. Importantly, this study had limitations: first, the samples analyzed were provided by voluntary participants; therefore, random sampling was not exercised. Second, the possibility of detecting substances is linked to the time of use, dose, and elimination half-life in urine before elimination; these factors were not analyzed in this study.

## Data availability statement

The original contributions presented in the study are included in the article/supplementary material, further inquiries can be directed to the corresponding author/s.

## Ethics statement

The studies involving human participants were reviewed and approved by Ethical Committee of Clinical Research of the Parc de Salut MAR (CEIC-PSMAR Number: 2018/8138/I) and Hospital Universitari Germans Trias i Pujol (CEIC-HUGTiP Number: PI-18-126). The patients/participants provided their written informed consent to participate in this study.

## Author contributions

MA-F, MT, and MF: formal analysis. MT, MF, RP, and SP: conceptualization, study design, funding acquisition, and supervision. MA-F, MP, EM, MR, RP, SP, CP-M, RM, EP, FF, MT, and MF: investigation. MA-F, MP, RP, SP, MT, and MF: methodology. RP, MT, and MF: project administration. MA-F, SP, MT, and MF: writing–original draft. All authors writing–review and editing and read and agreed to the published version of the manuscript.

## Funding

This work was supported by: European Union's Justice Programme—Drugs Policy Initiatives (number 806996—JUSTSO—JUST-2017-AG-DRUG) (GRANT AGREEMENT NUMBER: 806996-JUSTSO); Instituto de Salud Carlos III (ISCIII, Fondo de Investigación en Salud (FIS)-Fondo Europeo de Desarrollo Regional (FEDER), Grant Numbers: FIS PI17/01962 and FIS PI20/00804; Grants RD21/0009/0001 and RD21/0009/0004 funded by Instituto de Salud Carlos III (ISCIII-FIS-FEDER Red de Investigación en Atención Primaria de Adicciones-RIAPAd-RICORS), and by the European Union NextGenerationEU, Mecanismo para la Recuperación y la Resiliencia (MRR), Gencat Suport Grups de Recerca, Grant Numbers: 2017 SGR 316 and 2017 SGR 530; The Presidency of the Ministers Council, Department of Antidrug Policies in Italy.

## Conflict of interest

The authors declare that the research was conducted in the absence of any commercial or financial relationships that could be construed as a potential conflict of interest.

## Publisher's note

All claims expressed in this article are solely those of the authors and do not necessarily represent those of their affiliated organizations, or those of the publisher, the editors and the reviewers. Any product that may be evaluated in this article, or claim that may be made by its manufacturer, is not guaranteed or endorsed by the publisher.

## Abbreviations

NPS: new psychoactive substance
OUD: opioid use disorder
SC: synthetic cannabinoid
THC: tetrahydrocannabinol.
